# Glucocorticoids Downregulate PD-L1 in Glioblastoma Cells via GILZ-Mediated ERK Inhibition

**DOI:** 10.3390/biomedicines13081793

**Published:** 2025-07-22

**Authors:** Sabrina Adorisio, Giorgia Renga, Domenico Vittorio Delfino, Emira Ayroldi

**Affiliations:** 1 Section of Pharmacology, Department of Medicine and Surgery, University of Perugia, 06129 Perugia, Italy; adorisiosabrina@libero.it (S.A.); domenico.delfino@unipg.it (D.V.D.); 2 Foligno Nursing School, Department of Medicine and Surgery, University of Perugia, 06129 Perugia, Italy; giorgia.renga@unipg.it

**Keywords:** dexamethasone, GILZ, glioblastoma, glucocorticoids, immune checkpoints, immunotherapy, PD-L1

## Abstract

Glucocorticoids (GCs), such as dexamethasone (DEX), are commonly administered to glioblastoma (GBM) patients to control cerebral edema; however, their effects on immune checkpoint regulation in tumor cells remain insufficiently characterized. This study examined the impact of DEX on the expression of programmed death-ligand 1 (PD-L1) and glucocorticoid-induced leucine zipper (GILZ), a downstream effector of glucocorticoid receptor (GR) signaling, in the U87 and U251 glioblastoma cell lines. DEX consistently induced GILZ expression in both models yet elicited divergent effects on PD-L1: suppression in U87 cells and upregulation in U251 cells. In U87 cells, DEX-induced PD-L1 downregulation was accompanied by accelerated cell cycle progression, suggesting a dual impact on tumor immune evasion and proliferation. Mechanistically, GILZ silencing restored ERK phosphorylation and reversed PD-L1 suppression, whereas GILZ overexpression further decreased PD-L1 levels, implicating a GILZ–ERK pathway in the control of PD-L1. These findings uncover a previously unrecognized GR–GILZ–PD-L1 regulatory axis in glioblastoma cells. While these results are based on in vitro models, they provide a rationale for future in vivo studies to determine whether modulation of GILZ may influence immune checkpoint dynamics and therapeutic responsiveness in GBM.

## 1. Introduction

Programmed cell death protein 1 (PD-1) is an inhibitory receptor expressed on T cells that plays a crucial role in maintaining immune homeostasis by limiting excessive immune responses and promoting peripheral tolerance [1]. Upon binding to its ligand, PD-L1—expressed on both antigen-presenting cells and tumor cells—PD-1 signaling suppresses T cell receptor (TCR)-mediated activation, leading to reduced cytokine production, the inhibition of T cell proliferation, and impaired cytotoxic function [2]. This immune checkpoint mechanism is essential to prevent autoimmunity but is frequently exploited by tumors to evade immune surveillance [3]. In many cancers, the overexpression of PD-L1 facilitates immune escape by dampening antitumor T cell responses.

The blockade of the PD-1/PD-L1 axis with monoclonal antibodies has revolutionized cancer therapy [4], particularly in melanoma [5], non-small cell lung carcinoma [6], and renal cell carcinoma [7]. Immune checkpoint inhibitors (ICIs) targeting PD-1 or PD-L1 restore T cell effector function and have shown durable responses in a subset of patients [8]. However, resistance to ICIs and variability in treatment efficacy remain major clinical challenges [9,10,11,12].

In cancer patients, glucocorticoids (GCs) are administered as part of chemotherapy regimens, for symptom control, and to manage immune-related adverse events during ICI therapy [13]. Despite their clinical utility, the impact of GCs as antitumor agents remains controversial, due to their potential to interfere with immune checkpoint pathways, tumor cell biology, and the overall antitumor immune response [14,15].

A key molecular mediator of GC activity is the Glucocorticoid-Induced Leucine Zipper (GILZ), a transcriptional target of the glucocorticoid receptor (GR) [16,17]. The GILZ and its isoform, long-GILZ (L-GILZ) [18], exert potent anti-inflammatory and immunosuppressive effects by modulating NF-κB [19], MAPK, and AKT signaling pathways [20]. More recently, GILZ has also been implicated in tumor biology, influencing cell proliferation, apoptosis, and immune evasion [21,22,23,24].

Emerging evidence suggests a context-dependent relationship between GC exposure and PD-L1 expression [15]. In certain cancers, GCs can upregulate PD-L1 via GR-mediated transcriptional mechanisms, contributing to immune suppression and resistance to immunotherapy. Conversely, in other contexts, including specific glioma models, GC treatment may lead to PD-L1 downregulation, potentially restoring antitumor immune responses [25,26,27,28].

Glioblastoma (GBM) is the most aggressive and prevalent malignant brain tumor in adults, characterized by rapid proliferation, diffuse infiltration, treatment resistance, and a dismal prognosis, with median survival around 15 months despite standard therapies [29,30,31,32]. The GBM microenvironment is profoundly immunosuppressive, enriched in regulatory T cells, myeloid-derived suppressor cells, and immunosuppressive cytokines [33,34,35]. Additionally, GBM cells frequently upregulate immune checkpoint molecules such as PD-L1, contributing to immune evasion and posing significant barriers to effective immunotherapy [36]. Clinical outcomes with ICIs in GBM have thus far been disappointing, likely due to the complex immune microenvironment and frequent use of GCs in this patient population [37,38].

Dexamethasone (DEX), a potent synthetic GC, is routinely administered to GBM patients to manage tumor-associated cerebral edema [39,40]. However, its molecular effects in glioblastoma—particularly regarding PD-L1 regulation—remain poorly understood. Given that GILZ is a known mediator of many GC-induced immunosuppressive effects, it is critical to explore whether GILZ may be involved in modulating PD-L1 expression in GBM.

To address this gap, we investigated the effect of DEX on PD-L1 and GILZ expression in two well-characterized human GBM cell lines, U251 and U87, which are frequently used as comparative models in glioblastoma research due to their distinct molecular and phenotypic profiles [41,42,43]. GBM is, in fact, a highly heterogeneous tumor, and different cell lines reflect this variability. Transcriptomic studies have shown that U87 cells exhibit a neural-like molecular profile, while U251 and U373 display mesenchymal features. These differences correspond to distinct behaviors in proliferation, invasiveness, and treatment response. Therefore, using multiple GBM cell lines, such as U87 and U251, provides a representative model to explore diverse cellular responses to pharmacological or molecular interventions [44].

Our findings reveal that while GCs induce GILZ expression in both glioma lines, PD-L1 downregulation was observed only in U87 cells, suggesting a differential regulatory mechanism that may be relevant for optimizing immunotherapeutic strategies in GBM.

## 2. Materials and Methods

### 2.1. Chemicals and Reagents

DEX (Sigma-Aldrich, Burlington, MA, USA); DMEM medium (Euroclone, Milan, Italy); fetal bovine serum (FBS), penicillin/streptomycin (Gibco, Waltham, MA, USA); RIPA buffer (Thermo Fisher Scientific, Waltham, MA, USA) with protease and phosphatase inhibitors (Roche, Basel, Switzerland); primary antibodies: anti-PD-L1, anti-GILZ, anti-phospho-ERK, and anti-ERK (Cell Signaling Technology, Danvers, MA, USA); anti-GAPDH (OriGene, Rockville, MD, USA); Lipofectamine 2000, siGILZ, and control siRNA (Thermo Fisher Scientific, Waltham, MA, USA).

### 2.2. Cell Cultures and Treatments

Human tumor glioblastoma lines U251 and U87 were cultured in Dulbecco’s Modified Eagle Medium (DMEM) (Sigma-Aldrich, Burlington, MA, USA) supplemented with 10% fetal bovine serum (FBS; Sigma, USA), 100 U/mL penicillin, 1000 U/mL streptomycin, 2 mM L-glutamine, sodium pyruvate, and non-essential amino acids. Cells were seeded at a density of 15,000 cells/cm^2^) [41]. Cells were maintained at 37 °C in a humidified incubator with 5% CO_2_ [41]. For experiments, cells were seeded at appropriate densities and treated with DEX at final concentrations of 10^−6^ M, 10^−7^ M, or 10^−8^ M for 24 or 48 h. These concentrations were chosen based on previous experimental data, where GILZ upregulation was consistently observed [17,21]. Untreated cells served as controls.

### 2.3. Western Blot Analysis

Following treatment, cells were lysed using RIPA buffer (Thermo Fisher Scientific, Waltham, MA, USA) supplemented with protease and phosphatase inhibitors (Roche, Basel, Switzerland). Total protein concentration was determined, and 30 μg of protein per sample was separated by SDS-PAGE and transferred to nitrocellulose membranes (Millipore, Billerica, MA, USA). Membranes were blocked in 5% non-fat dry milk in TBS-T (Tris-buffered saline with 0.1% Tween-20) and incubated overnight at 4 °C with the following primary antibodies: anti-PD-L1 (Cell Signaling Technology, Danvers, MA, USA), anti-GILZ (Santa Cruz Biotechnology, Dallas, TX, USA), anti-phospho-ERK, anti-ERK (Cell Signaling Technology, Danvers, MA, USA), and anti-GAPDH (OriGene, Rockville, MD, USA), used as a loading control. After incubation with appropriate HRP-conjugated secondary antibodies, bands were visualized using enhanced chemiluminescence (Millipore, Billerica, MA, U.S). Densitometric analysis was performed using ImageJ software (ImageJ 1.4), with protein levels normalized to GAPDH and expressed as fold change relative to control [45].

### 2.4. Flow Cytometry

Cell viability and cell cycle distribution were analyzed via flow cytometry to determine the DNA content of cell nuclei stained with PI. Cells were harvested, washed with PBS, and incubated in water containing 50 μg/mL PI for 30 min at 4 °C [46] Fluorescence was measured by flow cytometry using Coulter Epics XL-MCL (Beckman Coulter Inc., Brea, CA, USA) and analyzed by the FlowJo_V10 software.

### 2.5. GILZ Silencing

For GILZ knockdown experiments, U87 cells were transfected with 50 nM small interfering RNA targeting GILZ (siGILZ) or a non-targeting control siRNA using Lipofectamine 2000 (Thermo Fisher Scientific, Waltham, MA, USA), according to the manufacturer’s protocol. Cells were harvested 48 h post-transfection for protein extraction and analysis.

### 2.6. GILZ Overexpression via TAT-Fusion Protein

To achieve transient overexpression of GILZ in U87 GBM cells, we employed a recombinant fusion protein composed of GILZ fused to the GST–TAT carrier (GST–TAT–GILZ). The GST tag facilitated purification and detection, while the TAT domain enabled efficient intracellular delivery without the need for genetic modification. U87 cells were treated with 0.2, 2, or 20 μg/mL of GST–TAT–GILZ for 24 h. Control cells received equivalent doses of GST–TAT lacking the GILZ domain. After treatment, cells were lysed in RIPA buffer, and protein extracts were analyzed by Western blot to assess GILZ expression levels and downstream targets [20]. This method allowed for a functional assessment of GILZ overexpression under near-physiological conditions.

### 2.7. Statistical Analysis

Data are presented as mean ± standard error of the mean (SEM) from at least three independent experiments. Statistical analyses were performed using one-way ANOVA followed by Tukey’s post hoc test. A *p*-value < 0.05 was considered statistically significant. GraphPad Prism™ software (version 6.0; GraphPad Software Inc., San Diego, CA, USA) was used for all statistical calculations and data visualization.

## 3. Results

### 3.1. DEX Induces GILZ Expression but Differentially Regulates PD-L1 in GBM Cell Lines

To investigate the effects of DEX on PD-L1 and GILZ protein expression in glioblastoma, we treated two human glioblastoma cell lines—U251 and U87—with increasing concentrations of DEX (10^−6^ M, 10^−7^ M, and 10^−8^ M) for 24 and 48 h. Western blot analyses were performed to assess protein levels of PD-L1 and GILZ, with GAPDH used as a loading control. A densitometric quantification of PD-L1 normalized to GAPDH revealed distinct, cell line-specific responses to DEX.

**Figure 1:**
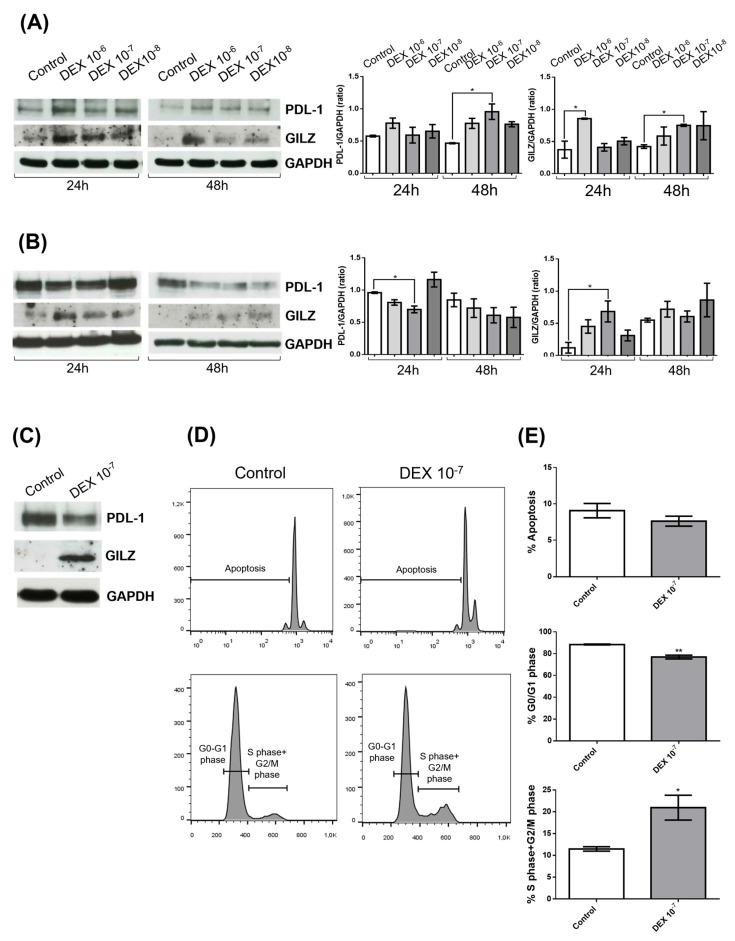
DEX modulates PD-L1 and GILZ expression and alters cell cycle distribution in glioblastoma cell lines. (**A**,**B**) Western blot analysis of PD-L1 and GILZ protein expression in U251 (**A**) and U87 (**B**) glioblastoma cells treated with DEX at concentrations of 10^−6^, 10^−7^, or 10^−8^ M for 24 or 48 h. GAPDH was used as a loading control. Representative immunoblots from three independent experiments are shown (left panels), with densitometric quantification of PD-L1 and GILZ levels shown on the right. Values were normalized to GAPDH and expressed as fold change relative to untreated controls. Data represent the mean ± SEM from three independent experiments. * *p* < 0.05 (one-way ANOVA). (**C**) Western blot analysis of PD-L1 and GILZ expression in U87 cells treated with 10^−7^ M DEX for 24 h. GAPDH was used as a loading control. (**D**) Representative flow cytometry histograms showing cell cycle distribution in U87 cells treated with 10^−7^ M DEX for 24 h, compared to untreated controls. The proportion of cells in G0/G1, S, and G2/M phases is indicated. (**E**) Quantification of apoptotic cells and the percentage of cells in G0/G1 and combined S + G2/M phases. Data represent the mean ± SEM from three independent experiments. * *p* < 0.05 and ** *p* < 0.01 (unpaired *t*-test).

In U251 cells (Figure 1A), DEX induced a dose-dependent increase in GILZ expression at both time points, consistent with GR activation. Notably, PD-L1 levels were also significantly upregulated at 48 h, especially at the DEX concentration 10^−7^, indicating a coordinated upregulation of both GILZ and PD-L1 in response to DEX.

In contrast, U87 cells (Figure 1B), displayed a divergent regulatory pattern. While DEX similarly upregulated GILZ in a dose-dependent manner, PD-L1 expression was significantly reduced, particularly at 24 h following treatment with 10^−7^ M DEX. This opposing trend in PD-L1 regulation—despite consistent GILZ induction—points to a cell line-specific modulation, likely influenced by differences in GR signaling dynamics, transcriptional co-regulators, or epigenetic landscape.

To further explore the functional relevance of the observed molecular changes, we focused on U87 cells treated with DEX (10^−7^ M for 24 h), a condition in which GILZ was upregulated while PD-L1 expression was reduced (Figure 1C). Flow cytometry analysis revealed that DEX treatment significantly altered cell cycle distribution. U87 cells displayed a reduced proportion in the G0/G1 phase and a corresponding increase in the S and G2/M phases indicative of accelerated cell cycle progression (Figure 1D,E). No significant differences in apoptosis were detected between treated and control cells.

Taken together, these findings demonstrate that DEX consistently induces GILZ expression in GBM cells but exerts opposing effects on PD-L1, enhancing it in U251 and repressing it in U87. Importantly, DEX promotes proliferation in U87 cells, as evidenced by altered cell cycle dynamics, while it does not affect proliferation in U251 cells.

### 3.2. GILZ Mediates PD-L1 Downregulation via ERK Inhibition in U87 Cells

To further investigate the inverse correlation between GILZ and PD-L1 expression observed upon DEX treatment, we modulated GILZ levels in U87 cells using both genetic silencing and protein-based overexpression strategies.

**Figure 2:**
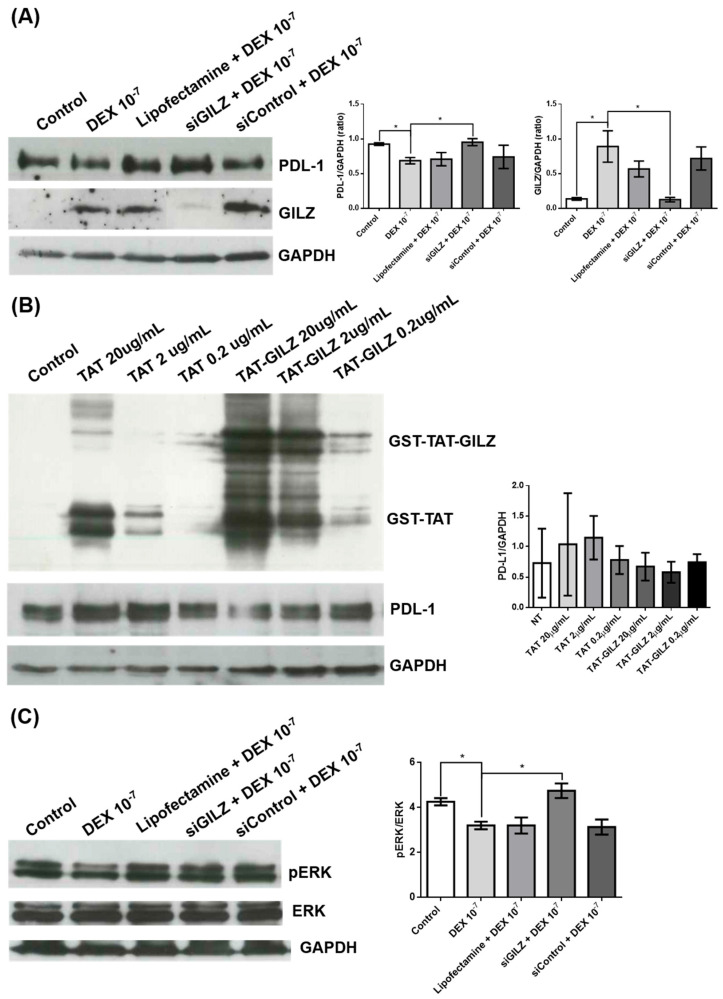
GILZ regulates PD-L1 expression and ERK signaling in U87 glioblastoma cells. (**A**) Western blot analysis of PD-L1 and GILZ protein levels in U87 cells under the following conditions: untreated (Control), treated with DEX (10^−7^ M), transfected with Lipofectamine alone, or transfected with GILZ-targeting siRNA (siGILZ) or control siRNA (siControl), both followed by DEX treatment. GAPDH was used as a loading control. Representative immunoblots from three independent experiments are shown (left panels) and densitometric quantification of PD-L1 and GILZ expression (normalized to GAPDH and expressed relative to control) is shown on the right. Data represent mean ± SEM. * *p* < 0.05 (one-way ANOVA). (**B**) U87 cells were treated with increasing concentrations of recombinant GST-TAT-GILZ fusion protein or GST-TAT control protein. Western blot analysis confirmed the presence of recombinant proteins and assessed PD-L1 expression. GAPDH was used as a loading control. Densitometric quantification of PD-L1 levels (normalized to GAPDH) is presented in the right panel. Data are expressed as mean ± SEM from three independent experiments. (**C**) Western blot analysis of total ERK and phosphorylated ERK (pERK) levels in U87 cells under the following conditions: untreated (Control), treated with DEX (10^−7^ M), transfected with Lipofectamine alone, or transfected with GILZ-targeting siRNA (siGILZ) or control siRNA (siControl), both followed by DEX treatment. Representative immunoblots from three independent experiments are shown (left panel). Densitometric quantification of the pERK/ERK ratio (normalized to total ERK and expressed relative to control) is shown in the right panel. Data are presented as mean ± SEM. * *p* < 0.05 (one-way ANOVA).

Western blot analysis showed that treatment with DEX (10^−7^ M) significantly increased GILZ expression and reduced PD-L1 protein levels. However, transfection with siGILZ markedly reduced GILZ expression and restored PD-L1 protein levels to those observed in untreated cells, despite DEX treatment. These results indicate that GILZ is required for the DEX-mediated repression of PD-L1. Control treatments with Lipofectamine and non-targeting control siRNA had no significant effect (Figure 2A). These findings demonstrate a causal role for GILZ in the regulation of PD-L1 expression in response to DEX.

To further confirm a direct role of GILZ in PD-L1 regulation, we treated U87 cells with a recombinant fusion protein consisting of GST-TAT-GILZ at two concentrations (2 and 0.2 µg/mL). GST-TAT-GILZ was efficiently internalized, as shown by the increased signal in the corresponding Western blot (Figure 2B). Although PD-L1 levels appeared progressively reduced with increasing concentrations of GST-TAT-GILZ, the trend did not reach statistical significance, likely due to variability and the limited number of replicates. GST-TAT alone had no effect, supporting the specific activity of the GILZ moiety. While preliminary, these results suggest that exogenous GILZ may contribute to PDL-1 downregulation.

Since ERK signaling has been implicated in the regulation of PD-L1 [47], we next investigated whether the GILZ-dependent PD-L1 modulation involved ERK pathway inhibition. Western blot analysis revealed that DEX treatment reduced ERK phosphorylation (as indicated by a decreased pERK/ERK ratio), and this effect was abrogated in siGILZ-transfected cells, indicating that GILZ mediates ERK pathway inhibition (Figure 2C) in this context.

In contrast, neither AKT phosphorylation nor p21 protein levels were affected by DEX treatment or GILZ silencing under the same experimental conditions (Supplementary Figure S1) suggesting a selective involvement of the ERK pathway in this regulatory mechanism. These data suggest that the downregulation of PD-L1 by GILZ may involve, at least in part, the suppression of ERK activation.

### 3.3. DEX Stimulates U87 Proliferation Independently of GILZ

**Figure 3:**
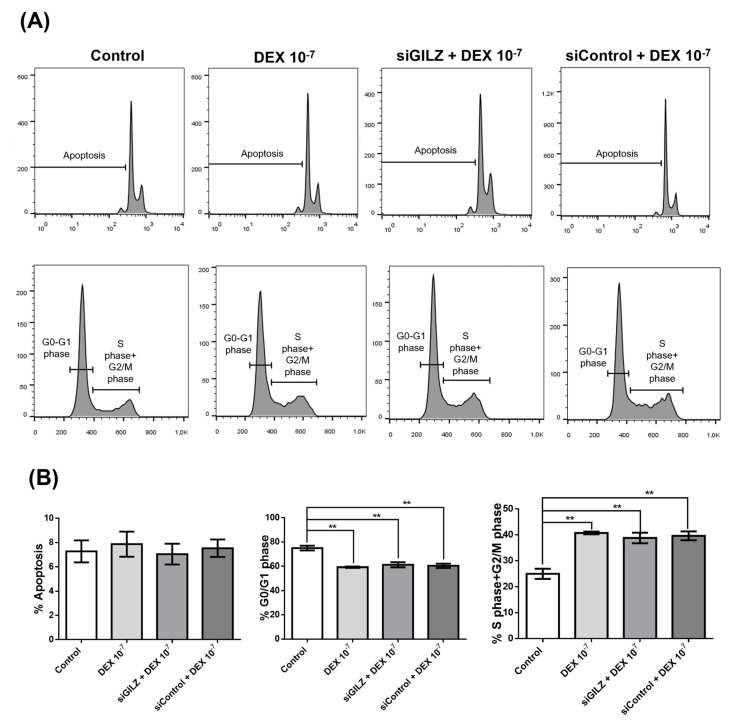
DEX-induced cell cycle progression in U87 cells is independent of GILZ. (**A**) Representative flow cytometry profiles showing apoptosis (**top panels**) and cell cycle distribution (**bottom panels**) in U87 cells under the following conditions: untreated (Control), treated with DEX (10^−7^ M for 24 h), treated with DEX and transfected with GILZ-targeting siRNA (siGILZ), and treated with DEX and transfected with control siRNA (siControl). Cell cycle histograms indicate the percentage of cells in G0/G1, S, and G2/M phases. (**B**) Quantification of apoptosis and distribution of cells in G0/G1 and S+G2/M phases. Data are presented as mean ± SEM from three independent experiments. ** *p* < 0.01 (one-way ANOVA with post hoc test).

To investigate whether GILZ mediates the proliferative effect of DEX in U87 GBM cells, we performed flow cytometric analysis of the cell cycle following GILZ silencing. Cells were left untreated, treated with DEX (10^−7^ M for 24 h), or transfected with siRNA targeting GILZ. This experimental setup was designed to assess proliferation under conditions in which DEX upregulates GILZ and downregulates PD-L1 (Figure 1C), to assess whether these molecular changes impact cell cycle dynamics. As shown in Figure 3A,B, DEX treatment significantly reduced the percentage of cells in the G0/G1 phase and increased the proportion of cells in S and G2/M phases, consistent with enhanced cell cycle progression. However, GILZ knockdown did not reverse these effects: siGILZ-transfected cells exhibited a cell cycle distribution nearly identical to that of DEX-treated controls, including the same increase in S+G2/M phase populations (Figure 3A). Apoptosis rates were unchanged across all conditions (Figure 3B).

These data indicate that the effect of DEX on cell cycle progression in U87 cells occurs independently of GILZ, suggesting that DEX may act through alternative signaling pathways, and that the modulation of PD-L1 and GILZ does not directly drive the proliferative response.

## 4. Discussion

This study uncovers a novel mechanism by which DEX, a synthetic GC widely used in oncology, modulates the expression of the immune checkpoint molecule PD-L1 in GBM cells via the induction of the GILZ protein. Notably, DEX consistently upregulated GILZ in both U87 and U251 GBM cell lines, but its effects on PD-L1 expression diverged—downregulating it in U87 and upregulating it in U251. This suggests that while GR activation and GILZ induction are conserved, PD-L1 regulation is influenced by cell-specific factors, such as chromatin accessibility, epigenetic status, or the presence of different transcriptional co-regulators.

Several studies have reported conflicting outcomes regarding the impact of GCs on PD-L1 expression across tumor types. In models of non-small cell lung cancer (NSCLC) and melanoma, GCs have been shown to upregulate PD-L1 expression, potentially contributing to immunosuppression and resistance to immune checkpoint inhibitors (ICIs) [48,49]. Conversely, other reports—particularly in glioma models—have observed PD-L1 downregulation in response to GR activation [50]. Our findings align with this latter scenario in U87 GBM cells, further supporting the context-dependent nature of GC action.

GILZ is a well-characterized downstream effector of GR signaling, known for its potent anti-inflammatory functions mediated through the inhibition of transcription factors such as NF-κB and AP-1 [16,19,20]. Recent evidence, however, has shown that GILZ can also influence cancer biology, exhibiting either pro- or anti-tumorigenic roles depending on the cellular context [51,52,53]. In GBM, our data suggest that GILZ negatively regulates PD-L1 expression, likely via the suppression of the ERK signaling pathway. This is consistent with previous reports that GILZ interacts with Ras/Raf components, leading to the suppression of ERK phosphorylation [20]. In our model, ERK inhibition correlates with PD-L1 downregulation, pointing to a potential mechanism by which GILZ may enhance anti-tumor immune responses.

Importantly, GILZ silencing abrogated both GILZ upregulation and ERK inhibition in DEX-treated U87 cells, supporting a causal role for GILZ in this pathway. However, GILZ silencing did not reverse DEX-induced changes in cell cycle progression—namely, the shift from G0/G1 into S and G2/M phases—suggesting that DEX also exerts GILZ-independent effects on GBM proliferation. These results highlight the selective role of GILZ in modulating immune-related pathways, while other proliferative effects of DEX are likely mediated through separate GR-dependent mechanisms.

This dual effect of DEX—reducing PD-L1 while simultaneously promoting proliferation—raises complex implications for its clinical use. While PD-L1 downregulation may sensitize tumors to immune responses or checkpoint blockade [6,36,49], enhanced cell cycle progression may paradoxically support tumor growth [54,55]. These opposing outcomes underscore the importance of dissecting GILZ-dependent and -independent pathways in GR signaling to better understand the net impact of glucocorticoid therapy in glioblastoma.

Interestingly, our findings diverge from those in dendritic cells, where GILZ silencing reduces PD-L1 expression and boosts their immunostimulatory activity [56,57]. In that setting, GILZ supports an immunosuppressive phenotype, indicating a positive role in PD-L1 regulation. This contrast likely reflects the distinct functional roles and signaling environments of immune versus tumor cells. In GBM, GILZ appears to act as a negative regulator of PD-L1, emphasizing the need to evaluate its effects in a cell type-specific manner.

It is important to note that these findings remain preliminary, as the mechanistic data are derived from a single glioblastoma cell line. Future studies using additional GBM models, including patient-derived and in vivo systems, will be crucial to validate the GILZ–PD-L1 regulatory axis and assess its potential relevance for glioblastoma immunotherapy.

## 5. Conclusions

Our study demonstrates that dexamethasone modulates PD-L1 expression in glioblastoma cells in a cell line-specific manner, with a concurrent and consistent induction of GILZ. In U87 cells, GILZ upregulation is associated with a reduction in PD-L1 expression and the inhibition of the ERK signaling pathway, suggesting a mechanistic link between GILZ and immune checkpoint regulation. Importantly, this effect occurs independently of changes in AKT phosphorylation or p21 expression. While these findings contribute to the understanding of glucocorticoid signaling in the glioblastoma microenvironment and highlight the potential of GILZ as a modulator of immunoregulatory pathways in cancer, it is important to note that the data are preliminary and based on in vitro analyses in a single cell line. Further validation in additional models and in vivo systems is required to confirm the relevance and generalizability of these observations.

## Data Availability

The data presented in this study, including raw western blot images, were submitted to the journal during peer review and are available upon request from the corresponding author.

## Supplementary Materials

The following supporting information can be downloaded at https://www.mdpi.com/article/10.3390/biomedicines13081793/s1. Figure S1. DEX treatment and GILZ silencing do not alter AKT phosphorylation or p21 expression in U87 glioblastoma cells. Western blot analysis of phosphorylated AKT (pAKT), total AKT, and p21 protein levels in U87 cells under the following conditions: untreated (Control), treated with DEX (10-M), transfected with Lipofectamine alone, or transfected with GILZ-targeting siRNA (siGILZ) or control siRNA (siControl), both followed by DEX treatment. GAPDH was used as a loading control. Representative immunoblots from three independent experiments are shown (left panel). Densitometric quantification of pAKT/AKT ratios and p21 levels (normalized to GAPDH and expressed relative to control) is shown (right panels). Data represent the mean ± SEM from three independent experiments. No statistically significant differences were observed (One-way ANOVA).

## Abbreviations

The following abbreviations are used in this manuscript:

DEX	Dexamethasone
GR	Glucocorticoid Receptor
PD-L1	Programmed Death-Ligand 1
GILZ	Glucocorticoid-Induced Leucine Zipper
siRNA	Small Interfering RNA
siGILZ	GILZ-targeting Small Interfering RNA
siControl	Control Small Interfering RNA
ERK	Extracellular Signal-Regulated Kinase
pERK	Phosphorylated Extracellular Signal-Regulated Kinase
AKT	Protein Kinase B
pAKT	Phosphorylated AKT
p21	Cyclin-Dependent Kinase Inhibitor 1A
GAPDH	Glyceraldehyde 3-Phosphate Dehydrogenase
SEM	Standard Error of the Mean
ANOVA	Analysis of Variance
