# Supplementary figures and images for: Glucocorticoids Downregulate PD-L1 in Glioblastoma Cells via GILZ-Mediated ERK Inhibition
Source: Biomedicines. 2025 Jul 22;13(8):1793. doi: 10.3390/biomedicines13081793 (PMC12383418; doi:10.3390/biomedicines13081793)

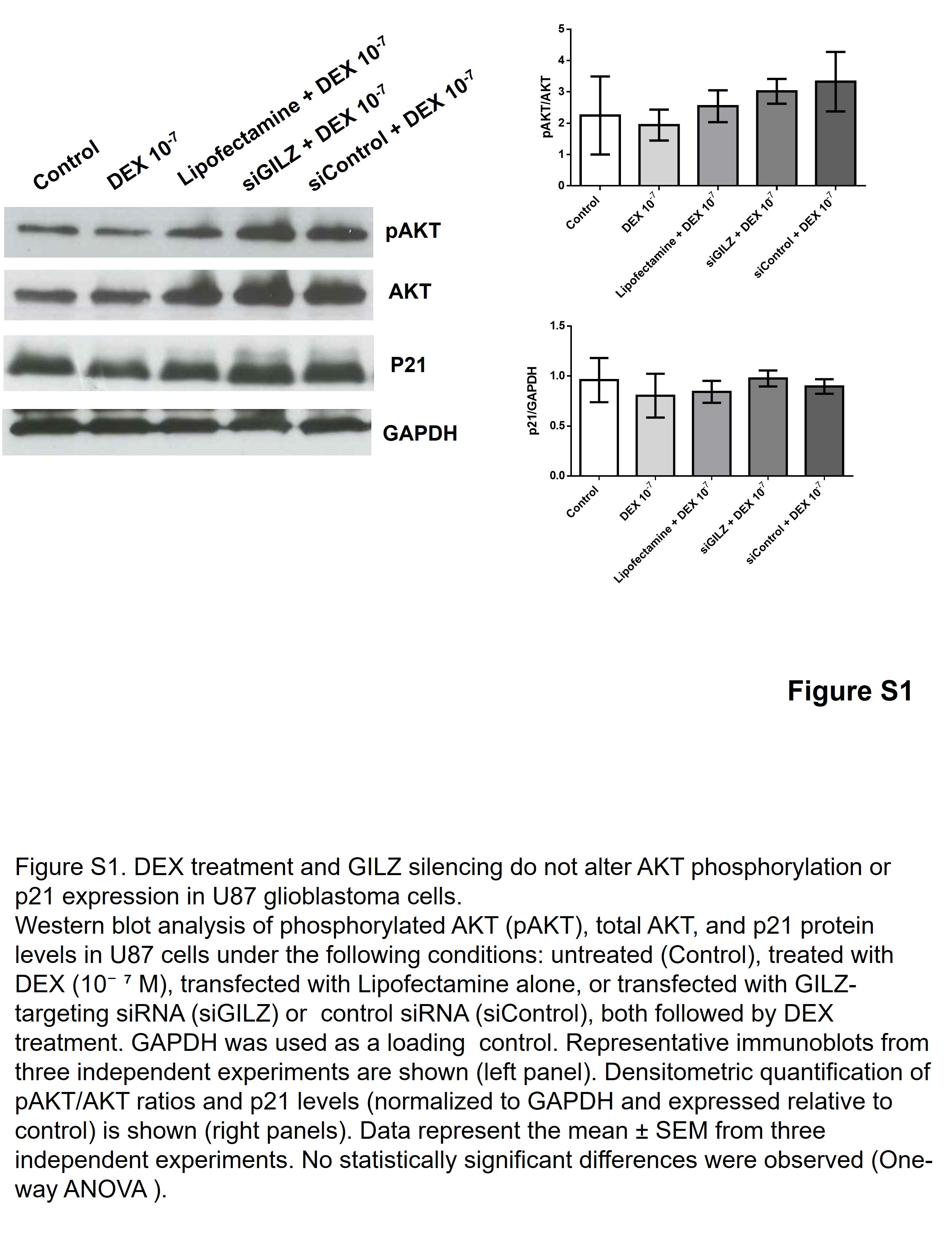

Supplement: Supplementary file 1 [file biomedicines-13-01793-s001.zip › Supplementary Figure S1.jpg]
